# Comparison of Two Methods of Antepartum Anticoagulation: Continuation of Enoxaparin until Scheduled Induction of Labor Versus Transitioning to Heparin with Spontaneous Labor

**DOI:** 10.1089/whr.2024.0039

**Published:** 2024-09-26

**Authors:** Marcia DesJardin, Edward Raff, Brian James, Angelina Mozier, Nicholas Baranco, Dimitrios Mastrogiannis

**Affiliations:** ^1^ Department of Obstetrics and Gynecology, SUNY Upstate, Syracuse, NY, USA.; ^2^ Booz Allen Hamilton, Baltimore, Maryland, USA.; ^3^ University of Maryland, Baltimore County, Maryland, USA.; ^4^ College of Medicine, SUNY Upstate, Syracuse, NY, USA.; ^5^ Department of Maternal-Fetal Medicine, SUNY Upstate, Syracuse, NY, USA.

**Keywords:** anticoagulation, labor, anesthesia

## Abstract

Pregnancy is a hypercoagulable state. There is a lack of strong evidence-based guidance regarding management when anticoagulation is required to prevent or treat venous thromboembolism during pregnancy. In practice, some patients are prescribed enoxaparin and transitioned to heparin due to the shorter half-life in the setting of an unpredictable delivery despite less predictable pharmacokinetics of heparin compared with enoxaparin, while others are continued on enoxaparin with a scheduled delivery. This work retrospectively evaluates obstetrical and neonatal outcomes between these two practices for 194 live singleton deliveries from 179 patients in a single institution January 2017 through May 2022. A Bayesian regression was used to control for confounders including dosing regimens. This work found no statistically significant differences in blood loss at time of delivery or availability of neuraxial anesthesia. This suggests continuing enoxaparin is noninferior to transitioning to heparin when anticoagulation is indicated in pregnancy.

## Objective

Pregnancy is a hypercoagulable state. Coagulation factors are increasingly produced while anti-thrombotic factors decline to accommodate the changing physiology required for gestation.^1–9^ As a result, there is approximately a fivefold increased risk of venous thromboembolism (VTE) in the antenatal and perinatal periods.^1,2^ In the presence of these additional risk factors, anticoagulation may be required. Mitigating the increased risk of maternal VTE while minimizing the risk to the developing fetus and morbidity during delivery poses a challenge.

The American College of Obstetricians and Gynecologists (ACOG) and Society for Maternal Fetal Medicine (SMFM) outline indications for antenatal anticoagulation. These recommendations include guidance for patients with a history of prior VTE, low-risk thrombophilia with a family or personal history of VTE, patients with high-risk thrombophilia, and patients with specific cardiac conditions such as mechanical heart valve.^5,6^ Based on the *a priori* risk of thromboembolism, ACOG has various dosing recommendations to accommodate the need for prophylactic or therapeutic anticoagulation.

To reduce risks to a developing fetus, heparin compounds are the preferred anticoagulants in pregnancy because none have been shown to cross the placenta.^7,8^ Unfractionated heparin and low molecular weight heparins, such as enoxaparin, are the most used heparin products with different advantages and disadvantages. Enoxaparin has more predictable pharmacokinetics with once daily dosing and equivalent efficacy to heparin. However, timing the cessation of anticoagulation is difficult to manage with the unpredictable nature of labor. Heparin requires more frequent dosing but has a shorter half-life and can be reversed in the event of spontaneous labor.^4,5,9^ There are demonstrated inconsistencies in laboratory values used to monitor the degree of heparin-induced anticoagulation [partial thromboplastin time (PTT) and antifactor Xa levels].^10^ Heparin has unpredictable pharmacokinetics in pregnant individuals due to changes in plasma volume, placental heparinase, and improved kidney filtration.^11^

Anticoagulation impacts obstetric care, including the availability of obstetric anesthesia. Neuraxial anesthesia may be utilized as an epidural in labor or as a spinal during a cesarean section. A waiting period is recommended based on the anticoagulation dose and medication used due to the risk of spinal hematoma with placement of neuraxial anesthesia,^9,12^ The Society for Obstetric Anesthesia and Perinatology (SOAP) recommends waiting 4–6 hours after 5,000 units of heparin twice or three times daily; 12 hours after enoxaparin 40 mg daily or 30 mg twice daily, or heparin 7,500–10,000 units twice daily; or 24 hours after any higher doses prior to attempting neuraxial anesthesia.^9^ If a cesarean section is necessary before this waiting period is complete, there becomes a dilemma of placement of neuraxial anesthesia or inducing general anesthesia. General anesthesia increases morbidity to both mother and baby for all patients, and is further increased in high-risk patients receiving venous thromboembolism prophylaxis. The SOAP guidelines allow for individual modification of above timeframes in urgent, high-risk cases. Nonetheless both anesthetic options have increased risk for the patient.

To mitigate the unpredictable nature of labor, SOAP recommends enoxaparin during early pregnancy and switching to heparin at 36 weeks.^9^ These recommendations are based on opinion. When therapeutic or intermediate dosing is required, switching to heparin requires close monitoring of clotting factors such as PTT and antifactor Xa levels due to the unpredictable pharmacokinetics.^10^ For prophylactic dosing, ACOG recommends 10,000 units heparin twice daily in the third trimester.^5^ This dose does not affect the SOAP recommended waiting time from that of enoxaparin. In contrast, the American Society of Hematology (ASH) recommends continuation of enoxaparin until delivery due to the unpredictable nature.^13^ Providers are faced with the conundrum of prescribing suboptimal dosing or a prolonged waiting period. There is a lack of strong evidence-based guidance regarding the specifics of management.^3–5,9^ When therapeutic dosing is required, there is a consensus favoring enoxaparin due to the predictability of the dosing of medication. In contrast, there remains unclear guidelines on which medication to use for prophylactic dosing.^5^

Maximizing the availability of neuraxial anesthesia can be a priority for some, which supports the switch to heparin. Switching anticoagulant agents is burdensome to both the patient and provider. Heparin has more unpredictable pharmacokinetics, making the transition to a therapeutic dose of heparin challenging. Additionally, the supplies are different for both medications, which creates a logistic and financial barrier.

In this work, we aim to evaluate maternal and fetal outcomes at time of delivery in pregnancies requiring antenatal anticoagulation at a single tertiary referral center.

## Materials and Methods

**FIG. 1.**
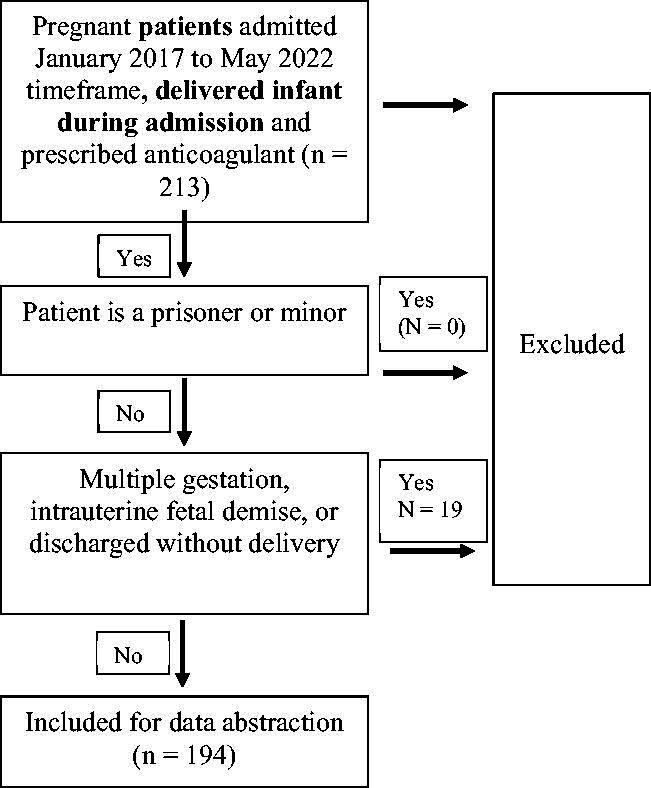
Inclusion and exclusion criteria used to determine the population studied.

Institutional review board approval was obtained (IRB No. 2021.0915, approved September 21, 2021). Charts for all patients who were admitted to a regional labor and delivery unit January 2017–May 2022, delivered during same admission, and were prescribed an antenatal anticoagulation at the time of admission *via* medication reconciliation process were reviewed. Patients were excluded if they met exclusion criteria: age less than 18 years old or incarcerated (Fig. 1). Patients who did not meet exclusion criteria and delivered a live singleton fetus during admission were included. Demographic, obstetric and delivery information were collected for each patient. The patients were placed into two groups: enoxaparin and heparin. Patients were placed in the enoxaparin group if the home medication admission reconciliation indicated the medication; it was assumed that enoxaparin was adhered to throughout the pregnancy. Alternatively, if a patient was prescribed heparin *via* home medication reconciliation, it was assumed that they either transitioned from enoxaparin at the end of the third trimester due to common practices or exclusively adhered to heparin throughout the pregnancy.

Our primary outcome is to evaluate differences in estimated blood loss at time of delivery between different anticoagulation strategies upon admission. Secondary outcomes include ability to receive neuraxial anesthesia, receipt of blood transfusions, and immediate fetal well-being.

To account for the multiple comparisons being performed, a Bayesian Logistic regression was performed against the prescribed anticoagulation (enoxaparin versus heparin) with the variables of interest and potential confounders included. In this way, one model is computed that can answer the significance of covariates *via* credible intervals that reduce likelihood of Type I errors^14^ and has been used in other medical work.^15^ The regression was performed using the Java Statistical Analysis Tool (JSAT) library.^16^ For comparison of population statistics *via* single variables, we critically note that this is used to determine study design but included only for descriptive qualities with multiple test correction.^17^ A non-parametric Welch test was used to account for the multiple data distributions that are not believed to be normal *via* application of a ranked order of values similarly to a Wilcoxoon–Mann–Whitney U-test.^18^ The non-parametric Welch test was used to avoid both false positive and negative errors due to misspecification, and such non-parametric testing is regarded as a better approach in hypothesis testing for medical applications.^19^

The potential confounders included in the Bayesian regression included: age, body mass index (BMI), number of previous deliveries, anticoagulation dosing (prophylactic, intermediate, or therapeutic), gestational age at delivery, whether labor was spontaneous or planned, if anticoagulation was continued in labor, concurrent sterilization at the time of delivery, delivery mode (vaginal versus cesarean, use of vacuum-assistance) due to the known impact on blood loss at the time of delivery.^20^

## Results

There were 194 live singleton deliveries from 179 patients meeting the criteria in this timeframe. Ninety-three patients were prescribed heparin prior to admission; 101 were prescribed only enoxaparin.

**FIG. 2.**
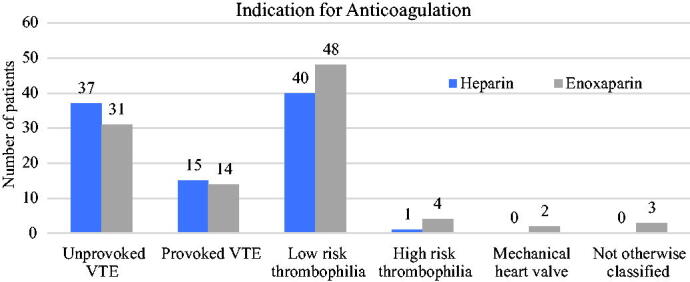
Indication for anticoagulation and frequency of heparin vs. enoxaparin use.

Indications for anticoagulation included unprovoked venous thromboembolism (37 prescribed heparin, 31 prescribed enoxaparin), provoked venous thromboembolism (15 heparin, 14 enoxaparin), low-risk thrombophilia such as heterozygous mutations or antiphospholipid syndrome (40 heparin, 48 enoxaparin), high-risk thrombophilia such as homozygous mutations (1 heparin, 3 enoxaparin), mechanical heart valve (0 heparin, 2 enoxaparin), or other (0 heparin, 3 enoxaparin) (Fig. 2). Patients were prescribed prophylactic dosing (80 heparin, 59 enoxaparin), therapeutic dosing (9 heparin, 28 enoxaparin), or intermediate dosing (4 heparin, 14 enoxaparin) (Table 1).

**Table 1.** Demographic, Obstetrical and Neonatal Information for Heparin versus Enoxaparin Groups. Statistical Significance is Based on the Logistic Regression. Continuous Variables Shown as Mean with Standard Deviation, Categorical Variables Shown as Number of Patients with Percentage of Group. NS = Not Significant; APGAR = Appearance, Pulse, Grimace, Activity, and Respiration scoring system

Variable	Heparin *N* = 93	Enoxaparin *N* = 101	Statistical significance
Demographic information			
Age (SD)	30.9 (±6.2)	30.2 (±6.1)	NS
Body mass index (SD)	33.2 (±7.4)	35.0 (±9.4)	NS
Number of previous deliveries (SD)	1 (±2)	1 (±1)	NS
Anticoagulation dosing			
Prophylactic dosing (%)	80 (86%)	59 (58%)	NS
Intermediate dosing (%)	4 (4%)	14 (14%)	NS
Therapeutic dosing (%)	9 (10%)	28 (28%)	NS
Delivery timing			
Gestational Age at Delivery in weeks (SD)	38.3 (±2.5)	37.5 (±2.7)	NS
Spontaneous labor (%)	24 (26%)	22 (22%)	NS
Scheduled induction of labor (%)	52 (56%)	45 (45%)	NS
Scheduled cesarean delivery (%)	17 (18%)	34 (34%)	NS
Anticoagulation continued in labor (%)	10 (11%)	11 (11%)	NS
Delivery details			
Concurrent sterilization (%)	8 (9%)	12 (12%)	NS
Vaginal delivery (%)	52 (56%)	50 (50%)	NS
Vacuum assisted delivery (%)	2 (2%)	10 (10%)	NS
Obstetrical outcome			
Estimated blood loss in mL (SD)	497 (±378)	491 (±311)	NS
Ability to receive neuraxial anesthesia (%)	66 (71%)	72 (71%)	NS
Birthweight in kg (SD)	3.185 (±0.675)	2.970 (±0.766)	NS
1-minute APGAR (SD)	7 (±2)	7 (±2)	NS
5-minute APGAR (SD)	9 (±1)	9 (±1)	NS
Neonatology intensive care unit (NICU) admission (%)	22 (24%)	28 (28%)	NS
Postpartum blood transfusion (%)	6 (6%)	3 (3%)	NS
Readmission for hematoma or bleeding	0	0	n/a
Reoperation	0	0	n/a
Immediate postpartum thromboembolism	0	0	n/a

Using a Bayesian regression that controls for differences in dosing, there were no statistically significant differences in patient characteristics between the enoxaparin only versus heparin groups (Table 1). There were no statistically significant differences in obstetrical or neonatal outcomes between the groups. This includes no statistically significant difference in our primary outcome of estimated blood loss at the time of delivery between the heparin group versus enoxaparin (Table 2) (497 mL ± 378 heparin versus 491 mL ± 311 enoxaparin).

**Table 2.** Logistic Regression Used to Compare Obstetrical Outcomes for Prophylactic versus Therapeutic and Intermediate Dosing. Known Confounding Variables Including Age, Body Mass Index, Number of Previous Deliveries, Continuation of Anticoagulation in Labor, Spontaneous vs. Induced Labor, Gestational Age at Time of Delivery, Assisted Second Stage of Labor, and Concurrent Sterilization. Credible Intervals Are Not Statistically Significant If Zero is Crossed

Variable	Prophylactic dosing (credible intervals)	Therapeutic/ intermediate dosing (credible intervals)
Estimated blood loss	(−142.413, 91.037)	(−196.338, 183.435)
Neuraxial anesthesia	(−1.187, 0.564)	(−0.903, 2.882)
Birth weight	(−0.255, 0.057)	(−0.241, 0.357)
Vaginal delivery	(−0.543, 0.989)	(−1.918, 1.284)
1 minute APGAR	(−0.235, 0.920)	(−1.401, 0.817)
5 minute APGAR	(−0.123, 0.460)	(−0.448, 0.33)

There was no statistically significant difference in any of the secondary outcomes between groups, including ability to receive neuraxial anesthesia (71% versus 71%), or blood transfusions (6% versus 3%) There were no statistically significant differences in immediate fetal outcomes including birthweight (3.185 kg ± 0.675 versus 2.970 ± 0.766), 1- or 5-minute APGARs (7,9 versus 7,9) or admission to the neonatology intensive care unit (24% versus 28%) (Table 2). There were no readmissions for bleeding, hematoma, wound infection, or reoperation.

A separate logistic regression was performed comparing dosing regimens. Prophylactic dosing was compared against the cohort of therapeutic and intermediate dosing regimens. The regression controlled for known confounding variables including age, body mass index, number of previous deliveries, if anticoagulation was continued in labor, spontaneity of labor, gestational age at time of delivery, vacuum assistance, and concurrent sterilization (Table 2). This logistic regression confirmed no statistical difference in primary and secondary outcomes between anticoagulation dosing method.

**FIG. 3.**
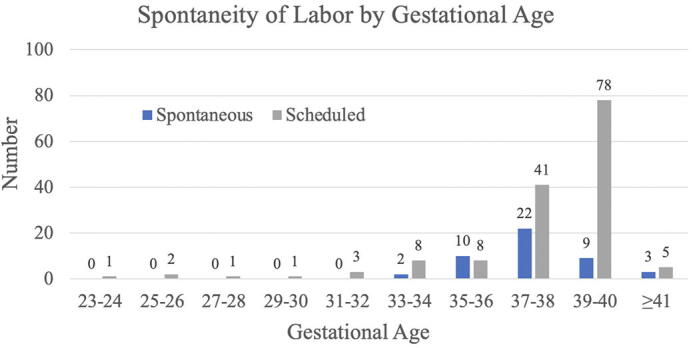
Number of patients who went into spontaneous labor by gestational age. Patients are commonly transitioned to heparin at 36 weeks. Of the 194 deliveries, 9 (4.6%) had spontaneous labor before 36 weeks. All patients who spontaneously labored less than 36 weeks were prescribed enoxaparin, and only one of five who had a cesarean section did not receive spinal anesthesia for the delivery.

**FIG. 4.**
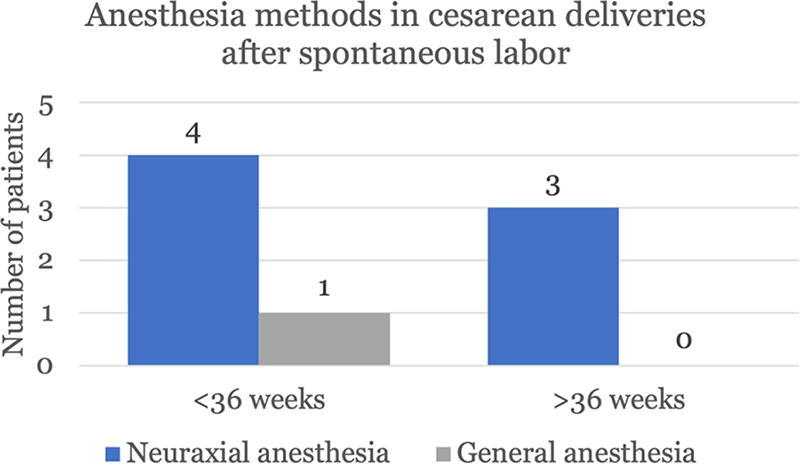
In patients who spontaneously labored while on enoxaparin, a small cohort underwent a cesarean section. Four of the five patients who spontaneously labored less than 36 weeks gestation received neuraxial anesthesia for the cesarean section. After 36 weeks gestation, all patients received neuraxial anesthesia if cesarean section was required.

Spontaneous labor was the minority of deliveries in this cohort. Forty-six of the 194 (24%) deliveries were spontaneous and 37 of the 46 (80%) occurred at or after 36 weeks gestation (Fig. 3). Of the patients prescribed enoxaparin, eight spontaneously labored and had a cesarean section. Five of spontaneous labors that resulted in cesarean section while on enoxaparin occurred before 36 weeks gestation, and four of the five (80%) were able to receive neuraxial anesthesia. The reason for general anesthesia in the one of five was because of severe thrombocytopenia secondary to HELLP syndrome at 33 weeks gestation. All the patients who spontaneous labored at or after 36 weeks while on enoxaparin and ultimately underwent a cesarean section received neuraxial anesthesia (Fig. 4).

## Discussion

In patients who require antenatal anticoagulation due to various pro-thrombotic risk factors, there are no statistically significant differences in obstetrical or neonatal outcomes between the practice of continuing enoxaparin until delivery versus transitioning to heparin in the late third trimester when adjusting for known risk factors. Spontaneous labor was less common in this cohort compared to scheduled delivery. Only one patient who spontaneously labored while on enoxaparin was unable to receive neuraxial anesthesia. This was due to thrombocytopenia in the setting of HELLP syndrome, not related to anticoagulation practice.

Despite the longer half-life of enoxaparin, the lack of statistically significant difference in outcomes suggests that it is clinically insignificant. These findings suggest spontaneous labor is prodromal enough to hold a dose prior to delivery. A held dose will decrease the anticoagulant concentration, which may contribute to the lack of difference seen.

There is no statistical difference in obstetrical outcomes between dosing regimens. Despite different degree of anticoagulation based on dosing, there is no difference in blood loss. This again may be secondary to the ability to withhold a dose of medication early enough in early labor to allow enough time to decrease active metabolites by the time of delivery.

Switching anticoagulants is burdensome due to changed dosing and the requirement to ascertain additional supplies required to transition to heparin. This work suggests there is utility in continuing enoxaparin until delivery.

This study is limited by the small sample size, including a particularly low sample size of patients on intermediate and therapeutic dosing. This may limit detection of statistically significant differences in bleeding outcomes. This is a retrospective review and reflects common practices at a single university tertiary center, which may limit its external validity to other locations utilizing other anticoagulation practices. Nevertheless, we believe that it reflects the safety of alternative anticoagulation practice. During the selected timeframe, cesarean deliveries transitioned from estimated blood loss to a measured quantitative blood loss. All blood loss for vaginal deliveries was estimated. The lack of consistency limits external validity. Patient collection was nonrandom as every patient who was prescribed anticoagulation in the timeframe and met both inclusion and exclusion criteria were included. Medication adherence was assumed among patients who included an anticoagulant on home medication reconciliation. The possibility of poor or no adherence to prescribed regimen limits internal validity.

Future directions of study include expanding review to include multiple labor and delivery units in multiple locations to verify the findings and increase its external validity. Future studies that characterize timing of last dose prior to delivery would help support the hypothesis that the lack of outcome difference is because patients are able to hold anticoagulation during early labor. Future prospective noninferiority trials will also be required to make practice-changing recommendations. Future pharmacokinetic studies may be of utility to validate heparin-monitoring laboratory values in pregnant patients.

## Abbreviations Used

ACOG: American College of Obstetricians and Gynecologists
APGAR: Appearance, Pulse, Grimace, Activity, and Respiration scoring system
ASH: American Society of Hematology
BMI: body mass index
JSAT: Java Statistical Analysis Tool
PTT: partial thromboplastin time
SMFM: Society for Maternal Fetal Medicine
SOAP: Society for Obstetric Anesthesia and Perinatology
VTE: venous thromboembolism

## References

[1] James AH. Pregnancy-associated thrombosis. Hematology Am Soc Hematol Educ Program 2009:277–285; doi: 10.1182/asheducation-2009.1.277 20008211

[2] Tepper NK, Boulet SL, Whiteman MK, et al. Postpartum venous thromboembolism: Incidence and risk factors. Obstet Gynecol 2014;123(5):987–996; doi: 10.1097/AOG.0000000000000230 24785851

[3] Pomp ER, Lenselink AM, Rosendaal FR, et al. Pregnancy, the postpartum period and prothrombotic defects: Risk of venous thrombosis in the MEGA study. J Thromb Haemost 2008;6(4):632–637; doi: 10.1111/j.1538-7836.2008.02921.x 18248600

[4] James AH, Jamison MG, Brancazio LR, et al. Venous thromboembolism during pregnancy and the postpartum period: Incidence, risk factors, and mortality. Am J Obstet Gynecol 2006;194(5):1311–1315; doi: 10.1016/j.ajog.2005.11.008 16647915

[5] James AH, Birsner M, Kaimal A. ACOG practice bulletin No. 196: Thromboembolism in pregnancy. 2018;132(4):1068; doi: 10.1097/AOG.0000000000002923 29939938

[6] Pacheco LD, Saade G, Shrivastava V, et al. Society for Maternal-Fetal Medicine (SMFM). Electronic address: pubs@smfm.org. Society for Maternal-Fetal Medicine Consult Series #61: Anticoagulation in pregnant patients with cardiac disease. Am J Obstet Gynecol 2022;227(2):B28–B43; doi: 10.1016/j.ajog.2022.03.036 35337804

[7] Bates SM, Middeldorp S, Rodger M, et al. Guidance for the treatment and prevention of obstetric-related venous thromboembolism. J Thromb Thrombolysis 2016;41(1):92–128; doi: 10.1007/s11239-015-1309-0 26780741 PMC4715853

[8] Greer IA, Nelson-Piercy C. Low-molecular-weight heparins for thrombprophylaxis and treatment of venous thromboembolism in pregnancy: A systemic review of safety and efficacy. Blood 2005;106(2):401–407; doi: 10.1182/blood-2005-02-0626 15811953

[9] Leffert L, Butwick A, Carvalho B, et al. Members of the SOAP VTE Taskforce. The society for obstetric anesthesia and perinatology consensus statement on the anesthetic management of pregnant and postpartum women receiving thromboprophylaxis or higher dose anticoagulants. Anesth Analg 2018;126(3):928–944; doi: 10.1213/ANE.0000000000002530 29099429

[10] McLaughlin K, Rimsans J, Sylvester KW, et al. Evaluation of Antifactor-Xa heparin assay and activated partial thromboplastin time values in patients on therapeutic continuous infusion unfractionated heparin therapy. Clin Appl Thromb Hemost 2019;25:1076029619876030; doi: 10.1177/1076029619876030 31530176 PMC6829967

[11] Brancazio LR, Roperti KA, Stierer R, et al. Pharmacokinetics and pharmacodynamics of subcutaneous heparin during the early third trimester of pregnancy. Am J Obstet Gynecol 1995;173(4):1240–1245; doi: 10.1016/0002-9378(95)91362-9 7485329

[12] Layton KF, Kallmes DF, Horlocker TT. Recommendations for anticoagulated patients undergoing image-guided spinal procedures. AJNR Am J Neuroradiol 2006;27(3):468–470. 16551977 PMC7976996

[13] Bates SM, Rajasekhar A, Middeldorp S, et al. American Society of Hematology 2018 guidelines for management of venous thromboembolism: Venous thromboembolism in the context of pregnancy. Blood Adv 2018;2(22):3317–3359; doi: 10.1182/bloodadvances.2018024802 30482767 PMC6258928

[14] Gelman A, Hill J, Yajima M. Why we (usually) don’t have to worry about multiple comparisons. Journal of Research on Educational Effectiveness 2012;5(2):189–211; doi: 10.1080/19345747.2011.618213

[15] DesJardin M, Raff E, Baranco N, et al. Cross-Sectional Survey of High-Risk Pregnant Women’s Opinions on COVID-19 Vaccination. Womens Health Rep (New Rochelle) 2022;3(1):608–616; doi: 10.1089/whr.2022.0006 35814609 PMC9258791

[16] Raff E. JSAT: Java statistical analysis tool, a library for machine learning. Journal of Machine Learning Research 2017;18(23):1–5.

[17] Forstmeier W, Wagenmakers EJ, Parker TH. Detecting and avoiding likely false-positive findings—a practical guide. Biological Reviews 2016; doi: 10.1111/brv.12315 27879038

[18] Delacre M, Lakens D, Leys C. Why psychologists should by default use Welch’s *t*-Test instead of Student’s t-Test. International Review of Social Psychology 2017;30(1):92–101; doi: 10.5334/irsp.82

[19] Kühnast C, Neuhäuser M. A note on the use of the non-parametric Wilcoxon-Mann-Whitney test in the analysis of medical studies. Ger Med Sci 2008;6:Doc02. 19675730 PMC2703264

[20] Wu E, Jolley JA, Hargrove BA, et al. Implementation of an obstetric hemorrhage risk assessment: Validation and evaluation of its impact on pretransfusion testing and hemorrhage outcomes. J Matern Fetal Neonatal Med 2015;28(1):71–76; doi: 10.3109/14767058.2014.905532 24670202

